# 

**Published:** 1866-01

**Authors:** 


CONTENTS.

ORIGINAL CONTRIBUTIONS.

Clinical Lectures on Diseases of the Eye. By E. L. Holmes, M. D......p. 1

The Hot Springs of Arkansas—Location—Character and Products of the Surrounding Country—Medicinal Qualities of the Waters, etc. By R. M. Lackey, M. D........ 4

New York Correspondence........ 11

SURGICAL CLINICS. By Dr. Edwin Powell. Reported by Dr. Lackey....... 14

Tumor on the Left Side of the Neck—Stone in the Bladder, 2 cases—Fistula in Ano.

ABSTRACTS AND SELECTED ARTICLES........ 18

*Anatomy and Surgery*—Strangulated Hernia treated successfully by Inflation of the Bowels—Bromine as an Antidote to Zymotic Poisons—U. S. A. Surgical Reports—An Operation for Fistula in Ano Two Hundred Years Ago.

*Practice of Medicine and Pathology*—Cholera: Is it Transmissible?

*Materia Medica and Therapeutics*—The Hypophosphites, their Therapeutic Value.

MISCELLANEOUS........ 30

A Remarkable Case of Double Monstrosity in an Adult—Literary Men and Doctors—Aneurism of the Sciatic Artery Treated by Injections of Perchlorure de Fer—Human Deterioration—Ventilation of Sewers—Trichinæ—Regeneration of Bone—Lemon Juice for Cancer.

EDITORIAL........ 37

Valedictory—Salutatory.

*Book Notices*—The Practice of Medicine and Surgery Applied to the Diseases and Accidents Incident to Women—Rhynoscopy and Laryngoscopy—Lectures on the Diseases of Infancy and Childhood—Sixth and Seventh Annual Report of the Chicago Charitable Eye and Ear Infirmary, etc., etc.

THE CHICAGO

